# 

**Published:** 1896-07-04

**Authors:** 


**Notices of Births, Deaths, and Marriages** are inserted in this column at a charge of 2s. 6d. for an announcement not exceeding four lines.

**Notice to Advertisers and Subscribers.**

All Advertisements, Orders for Copies of The Hospital and Business Communications should be addressed to **THE MANAGER, (Not to The Editor),** The Hospital, 428, Strand, London, W.C.

The Cost of Subscription, post free, is as follows:—Per week, 2½d; per quarter, 2s. 8d.; per half-year, 5s. 3d.; per annum, 10s. 6d. Foreign:—Per Annum, 15s. 2d.; payable, in all cases, in advance.

Just Published.

**"Burdett's Hospitals and Charities, 1896."**

Seventh year of publication. Over 1,000 pp. Scarlet cloth, gilt lettered, Price 5s. A most complete guide to British, Colonial, Indian, and American Hospitals, Dispensaries, Nursing and Convalescent Institutions, and Asylums. A few complete sets of the preceding six issues of the "Annual" may still be obtained on application to the Publishers, The Scientific Press (Limited), 428, Strand, London, W.C.

### NOTICE TO CORRESPONDENTS.

All MS., Letters, Books for Review, and other matters intended for the Editor should be addressed The Editor, The Lodge, Porchester Square, London, W.

The Editor cannot undertake to return rejected MS., even when accompanied by stamped directed envelope.

**NOTICE.—Vol XIX. of "The Hospital" (October, 1895, to April, 1896), in handsome cloth binding, is now on sale at the Office of this Journal. Price 7s. 6d. Cases for binding the Numbers contained in this Volume, 1s. 6d. Index to Vol. XIX., 2½d., post free. The Monthly Part for July is now ready, price 6d., by post 8d.**

### SCRAPS AND GLEANINGS.

The operating theatre at the Addenbrooke's Hospital, Cambridge, is to be improved.

Two new operating theatres are now in course of erection at the Leeds General Infirmary.

An exhibition on a large scale is to be held in 1897 at Newcastle in aid of the rebuilding of the Royal Infirmary.

The first Hospital Saturday in connection with the Chichester Infirmary was held on the 16th ult.

The Royal South Hants Infirmary is to be rebuilt and enlarged. The work is estimated to cost £19,000

A number of persons in Bradford are suffering through eating ice creams, and in one case—that of a boy—death has followed.

A wordy war had been raging at Exeter over the proposal of the Devon and Exeter Hospital to light the hospital with electric light.

During the year 1895, 175 in-patients and 2,216 out-patients have been treated at the Samaritan Hospital for Women, Nottingham.

The special appeal made by the Mayor has brought in sufficient funds to extinguish the debt incurred by the Stratford-upon-Avon Hospital.

An administration building and two hospital cottages are to be constructed for the Massachusetts Hospital for Epileptics, at Monson, Mass.

The trustees of Smith's Charity have given £100 to the General Fund of the Grosvenor Hospital for Women and Children, Vincent Square, Westminster.

It has been decided to omit the word "cottage" in the title of the St. Helens Cottage Hospital, so that in future it will be known as "The St. Helens Hospital."

A Leamington lady has left a legacy of £1,200 to the Warneford, Leamington, and South Warwickshire Hospital, in order to endow a bed for cancer patients.

The house-to-house canvass undertaken by the Ladies' Committee, formed by Mrs. Lucas-Shadwell to help the Hastings Hospital, has now been definitely arranged.

The number of patients attending at the Newcastle Eye Infirmary in 1895 amounted to 6,558. The total receipts amounted to £1,120, and the total payments to £1,100.

Messrs. Crosse and Blackwell and Mr. T. F. Blackwell have each contributed 100 guineas in aid of the new Seaside Convalescent Home of the Middlesex Hospital.

The committee appointed to consider the question of erecting a Home for Incurables at Dundee have appointed a sub-committee to prepare a scheme for future discussion.

A suggestion has been made in Coventry that each cycle manufacturer should set aside the sum of one penny on every machine turned out of the factory and give it to the hospital.

The offer of the Dowager Lady Tweedmouth to erect a memorial chapel in memory of her husband, for the use of the Northern Infirmary, Inverness, has been unanimously accepted.

The rummage sale held on the 13th ult. in aid of the Whitchurch Cottage Hospital produced nearly £77, of which £70 (the net proceeds) has been handed over to the institution.

At the annual meeting of the Glasgow Ear Hospital it was stated that at no distant date the directors would have to face the question of increased accommodation and increased staff.

The income of the Barnsley Hospital Saturday and Sunday Committee shows a slight falling off in 1895 when compared with 1894. The receipts were £1,045 in 1895 and £1,112 in 1894.

The receipts at West Bromwich on Hospital Saturday in aid of the West Bromwich District Hospital amounted to £515. The Oldbury Hospital Saturday Committee received in addition for the same institution £348.

The North Infirmary and City of Cork General Hospital, during 1895, had a daily average of 67 beds occupied—the same number as in 1894; and 7,247 accident cases were treated during the year, against 5,789 in 1894.

The Newcastle Hospital Sunday Fund has during the past year received £1,974 from collections in places of worship, and £2 088 from collections in manufactories. The total income amounted to £4,334, and the expenditure to £360.

Twenty-six years ago, *i.e.*, in 1870, 694 in-patients were admitted to the Bath Royal Mineral Water Hospital. Last year (1895) 1,240 were treated. The total accommodation of the hospital at these two periods was 145 and 171 beds respectively.

The report of the North Ormesby Cottage Hospital states that the deficit in the funds still delays the important work of remodelling the kitchen and domestic offices of the hospital, which were originally built to accommodate thirty in-patients, and have now to serve for double that number.

At the last annual meeting of the Kidderminster Infirmary the working men's representatives were debarred from voting, as the rules then in force did not give such representatives the power to vote. The rules have now been revised, and a special meeting has been held at which they were unanimously agreed to.

The public elementary schools of Bristol, and the Foresters in that neighbourhood, each subscribed £50 (the amount requisite to support a cot for a year) to the Convalescent Home for Children at Weston-super-Mare, the seaside branch of the Bristol Children's Hospital. The balance sheet shows that £115 had to be provided by the parent institution to make up the deficiency in the income of the home in 1895.

The sum promised towards the erection of the new hospital at Newport, Mon., amounts to about £7,000, exclusive of the offer of Dr. Garrod Thomas to give a donation equal to half the amount collected. The directors have determined not to proceed with the matter of plans until the donations amount to £10,000, exclusive of Dr. Thomas's £5,000. The site (three acres) has been given by Lord Tredegar, and the buildings are expected to cost about £16,000 or £17,000, exclusive of furnishing.

### The Student of Medicine.

### APPOINTMENTS.

R. T. Hugh, L.R.C.P.Lond., M.R.C.S., appointed Medical Officer of the Workhouse and Schools of the Birkenhead Union. W. H. A. Jacobson, M.B., F.R.C.S.Eng., appointed Examiner in Anatomy for the Fellowship of the Royal College of Surgeons of England. C. B. Ker, M.D., appointed Resident Medical Officer to the Edinburgh Fever Hospital. A. H. N. Lewers, M.D.Lond., M.R.C.S.Eng., appointed Examiner in Midwifery for the Third Examination of the Royal College of Surgeons of England. C. L. Lightfoot, M.D., C.M.Edin., M.R.C.S.Eng., appointed Surgeon to the Northumberland, Durham, and Newcastle Eye Infirmary. C. D. Lindsey, M.R.C.S., L.R.C.P., appointed House Surgeon to the Horton Infirmary, Banbury. C. B. Lockwood, F.R.C.S.Eng., appointed Examiner in Anatomy for the Fellowship of the Royal College of Surgeons of England. B. T. Lowne, F.R.C.S.Eng., appointed Examiner in Physiology for the Fellowship of the Royal College of Surgeons of England. R. C. Lucas, M.B., B.S.Lond., F.R.C.S.Eng., appointed Examiner in Anatomy for the Second Examination of the Royal College of Surgeons of England. A. P. Luff, M.D.Lond., M.R.C.S., appointed Examiner for Diploma in Public Health of the Royal College of Surgeons of England. G. H. Makins, F.R.C.S.Eng., appointed Examiner in Anatomy for the Second Examination of the Royal College of Surgeons of England. A. Morison, M.D., F.R.C.P.Edin., M.R.C.P.Lond., appointed Physician to Out-patients at the Children's Hospital, Paddington Green. P. Paget, L.R.C.P., M.R.C.S., appointed Medical Officer for the High Halden District of the Tenterden Union. D'Arcy Power, M.B., F.R.C.S.Eng., appointed Examiner in Physiology for the Second Examination of the Royal College of Surgeons of England. C. S. Prideaux, L.D.S.Eng., appointed Honorary Dental Surgeon to the Dorset County Hospital, Dorchester. P. Rankin, L.F.P.S.Glasg., appointed Medical Officer for the Third District of the First Division of Greystoke of the Penrith Union. A. Routh, M.D., B.S.Lond., M.R.C.S.Eng., appointed Examiner in Midwifery for the Third Examination of the Royal College of Surgeons of England.

### SOCIETY OF APOTHECARIES OF LONDON.

The following is a list of the examiners of the Society of Apothecaries of London for 1896-7, showing the subjects on which they examine and the London medical schools to which they are respectively attached as teachers. The examiners in medicine are all Fellows of the Royal College of Physicians of London, and the examiners in surgery are all Fellows of the Royal College of Surgeons of England:—

Medicine.—*Sir H. R. Beevor, M.D.Lond., F.R.C.P.Lond., King's College; *C. Y. Biss, M.D.Cantab., F.R.C.P.Lond., Middlesex Hospital; *F. de Havilland Hall, M.D.Lond., F.R.C.P.Lond., L.S.A., Westminster Hospital; *A. P. Luff, M.D.Lond., F.R.C.P.Lond., St. Mary's Hospital; G. Newton Pitt, M.D.Cantab., F.R.C.P.Lond., Guy's Hospital; *H. D. Rolleston, M.D.Cantab., F.R.C.P.Lond., St. George's Hospital; *Francis Warner, M.D.Lond., F.R.C.P.Lond., L.S.A., London Hospital.

Surgery.—Appointed by the Medical Council: *W. Adams Frost, F.R.C.S.Eng., St. George's Hospital; *Arthur T. Norton, F.R.C.S.Eng., St. Mary's Hospital; *Bernard Pitts, F.R.C.S.Eng., St. Thomas's Hospital; *Bilton Pollard, F.R.C.S.Eng., University College; *C. B. Lockwood, F.R.C.S.Eng., St. Bartholomew's Hospital. Appointed by the Society: L. A. Dunn, F.R.C.S.Eng., Guy's Hospital.

Midwifery.—Wm. John Gow, M.D.Lond., St. Mary's Hospital; *Arthur H. N. Lewers, M.D.Lond., L.S.A., London Hospital.

Anatomy.—F. G. Parsons, F.R.C.S.Eng., St. Thomas's; J. W. S. Boyd, M.B.Lond., F.R.C.S.Eng., Charing Cross.

Physiology and Biology.—P. T. Beale, F.R.C.S.Eng., King's College; H. W. M. Tims, M.D., M.S.Edin., Westminster Hospital.

Chemistry.—H. Forster Morley, M.A., D.Sc.Lond., Charing Cross Hospital; T. J. M. Page, B.Sc.Lond., London Hospital.

Materia Medica and Pharmacy.—H. Sainsbury, M.D.Lond., F.R.C.P.Lond., Royal Free Hospital; J. Galloway, M.D., Aberdeen, London Hospital.

*Marked thus members of the Court of Examiners.

